# What non-pharmacological treatments do people with polymyalgia rheumatica try: results from the PMR Cohort Study

**DOI:** 10.1007/s00296-021-05036-6

**Published:** 2021-10-22

**Authors:** Jake Weddell, Samantha L. Hider, Christian D. Mallen, Sara Muller

**Affiliations:** 1 School of Medicine, Keele University, Keele, Staffordshire, UK; 2 Haywood Academic Rheumatology Centre, Midlands Partnership NHS Foundation Trust, Stoke-on-Trent, UK

**Keywords:** Polymyalgia rheumatica, Exercise, Diet, Complementary therapies, Surveys and questionnaires

## Abstract

Polymyalgia rheumatica (PMR) is common. The mainstay of treatment, glucocorticoids, are associated with significant adverse effects and many patients remain on high doses for a number of years. Little is known about the use of other, non-pharmacological therapies as adjuncts in PMR. The PMR Cohort Study is an inception cohort study of patients diagnosed with PMR in primary care. This analysis presents data on the use and perceived impact of non-pharmacological therapies from a long-term follow-up survey. Non-pharmacological treatments were classified as either diet, exercise, or complementary therapies. Results are presented as adjusted means, medians, and raw counts where appropriate. One hundred and ninety-seven participants completed the long-term follow-up questionnaire, of these 81 (41.1%) reported using non-pharmacological therapy. Fifty-seven people reported using a form of complementary therapy, 35 used exercise and 20 reported changing their diet. No individual non-pharmacological therapy appeared to be associated with long-term outcomes. The use of non-pharmacological therapies is common amongst PMR patients, despite the paucity of evidence supporting their use. This suggests that people perceive a need for treatment options in addition to standard glucocorticoid regimens. Further research is needed to understand patients’ aims when seeking additional treatments and to strengthen the evidence base for their use so that patients can be guided towards effective options.

## Introduction

Polymyalgia rheumatica (PMR) is an inflammatory condition, which is characterised by pain and stiffness in the shoulder and hip girdles, predominantly affecting those aged over 50 years. Additional symptoms include fatigue, fever, anorexia, and weight loss; often accompanied by a rise in inflammatory markers. PMR is a common condition, with a lifetime prevalence estimated to be 0.85% in the UK [1]. Symptoms can cause significant disability and substantially reduce quality of life [2, 3].

Glucocorticoid medication is the mainstay of PMR treatment [4]; however, prolonged use is associated with significant adverse effects. Most treatment regimens for PMR consist of an initial dose of glucocorticoids to induce remission, followed by a tapering regimen to minimise total glucocorticoid exposure. Relapses are common during the tapering period. Whilst there are few evidence-based additional treatment options beyond the use of steroids sparing agents, such as methotrexate [5], many patients remain on glucocorticoid therapy for a significant length of time. Given the challenges of long-term glucocorticoid treatment, patients are often keen to explore other potential treatment options [6, 7]. However, studies exploring use of non-pharmacological therapies for PMR are sparse, particularly in comparison to other rheumatological conditions. A recent systematic review into non-pharmacological therapies for rheumatoid arthritis included 91 randomised controlled trials and 9 observational studies [8]. In comparison the EULAR/ACR PMR collaborative [9] identified just 2 studies that considered herbal medicine for PMR and no controlled studies into non-pharmacological therapies. Despite the paucity of evidence, the collaborative did recommend, “considering an individualised exercise programme for PMR patients,” although there were no studies of exercise or physiotherapy for people with PMR. Given the benefits of exercise for maintaining muscle mass and function and reducing falls risk, this recommendation may be understandable, but currently there is no evidence base to support implementation which may prevent services being commissioned.

The PMR Cohort Study assessed the epidemiology and long-term outcomes of people diagnosed with PMR in primary care [10]. The aim of this analysis was to investigate the reported use and perceived benefit of non-pharmacological and exercise therapies for PMR and the association of these therapies with long-term outcomes.

## Methods

### Study design

The PMR Cohort Study has been described in detail elsewhere [10]. Briefly, participants were recruited from 382 GP practices across England, between June 2012 and June 2014. Eligible patients were flagged to the clinician when PMR was entered into their record during a consultation, using electronic prompts in the primary care record. Patients who verbally consented were sent a study pack including participant information sheet, baseline questionnaire and consent form. Responders to this questionnaire were followed up via regular self-completion questionnaire up to 24 months. Patients who had not withdrawn from the study at this stage were then sent a long-term follow-up questionnaire (LTFU) between January and June 2019.

### Data collection

Patients were included in this analysis if they responded to both the baseline and the LTFU questionnaire. 197 participants completed both the baseline and the LTFU questionnaire. Responders and non-responders to the LFTU questionnaire were broadly similar in terms of their sociodemographic and PMR characteristics at baseline, although responders tended to live in less deprived areas. An analysis of the attrition from the cohort up to 2 years has previously been reported [11] and attrition from the LTFU will be published separately.

The LTFU collected information pertaining to use of non-pharmacological treatments, and the perceived helpfulness of these treatments using a patient completed checklist. Treatments were grouped as exercise (which included an increase in exercise and hydrotherapy), complementary therapies (which included acupuncture, Alexander technique, aromatherapy, herbal medicine, homeopathy, massage and, vitamin and mineral supplementation) and diet or weight loss. In addition, the questionnaire asked participants to record whether exercises specifically for PMR were advised and from whom the patient received this advice (general practitioner, hospital physician, or physiotherapist, with the option to check more than one box).

Data on PMR outcomes were extracted from baseline and LTFU questionnaires and included pain and stiffness measured by a numerical rating scale (from none 0–10 as bad as can be), whether the patient could lift their arms above their head and daily prednisolone dose. At LTFU participants were asked whether their PMR symptoms had improved since first presentation (completely recovered, much improved, or improved). Additionally, the number of relapses was extracted from the LTFU.

### Statistical analysis

Non-pharmacological therapies used are presented as *n* (%). Sociodemographic data are presented as means and standard deviations, medians and interquartile ranges or *n* (%) as appropriate. Outcomes are presented as either adjusted means, with the related baseline variable as the adjusting factor, or as *n* (%). Adjusted means were computed using one-way ANCOVA and are presented with 95% confidence intervals. Analysis was performed using SPSS v27.

## Results

Overall, 197 patients completed both questionnaires. The mean age at diagnosis was 72.0 (SD 8.2) and 63.5% were female (Table 1). Of the 197 participants, 81 (41.1%) reported use of a non-pharmacological treatment during their disease course. People reporting use of non-pharmacological treatments were younger (69.4 vs 73.8) and more likely to be female (74.1% vs 56.0%), although the baseline symptom reporting between both groups was similar (Table 1).

**Table 1:** Demographics and baseline features of users and non-users of non-pharmacological therapies

Demographics	No therapy (*n* = 116)	Any therapy (*n* = 81)	Increased exercise (*n* = 35)	Diet/weight loss (*n* = 20)	Complementary therapy (*n* = 57)	Recommended exercise (*n* = 50)
Age at diagnosis [mean(SD)]	73.8 (7.4)	69.4 (8.7)	68 (9.9)	70.2 (9.6)	69.7 (8.4)	70.3 (7.8)
Female [*n* (%)]	65 (56.0%)	60 (74.1%)	28 (80%)	15 (75.0%)	44 (77.2%)	30 (60.0%)
BMI at baseline [mean(SD)]	26.7 (4.1)	28.4 (6.3)	27.6 (4.1)	32.1 (8.2)	28.4 (5.9)	28.4 (6.8)
Baseline prednisolone dose [mean(SD)]	15.3 (8.0)	16.1 (6.9)	15.9 (7.2)	15.8(7.4)	16.3 (7.2)	15.9 (8.1)
Baseline pain NRS [median(IQR)]	8.0 (7.0–10.0)	8.0 (7.0–9.0)	8.0 (6.0–9.0)	8.0 (6.3–9.0)	8.0 (7.0–9.0)	8.0 (6.8–9.0)
Baseline stiffness NRS [median(IQR)]	8.0 (7.0–9.0)	8.0 (6.5–9.0)	7.0 (6.0–9.0)	8.0 (5.3–10.0)	8.0 (7.0–9.0)	8.0 (7.0–9.0)
Baseline morning stiffness > 1 h [*n* (%)]	86 (74.1%)	55 (67.9%)	24 (68.6%)	13 (65.0%)	39 (68.4%)	38 (76.0%)

**Fig. 1:**
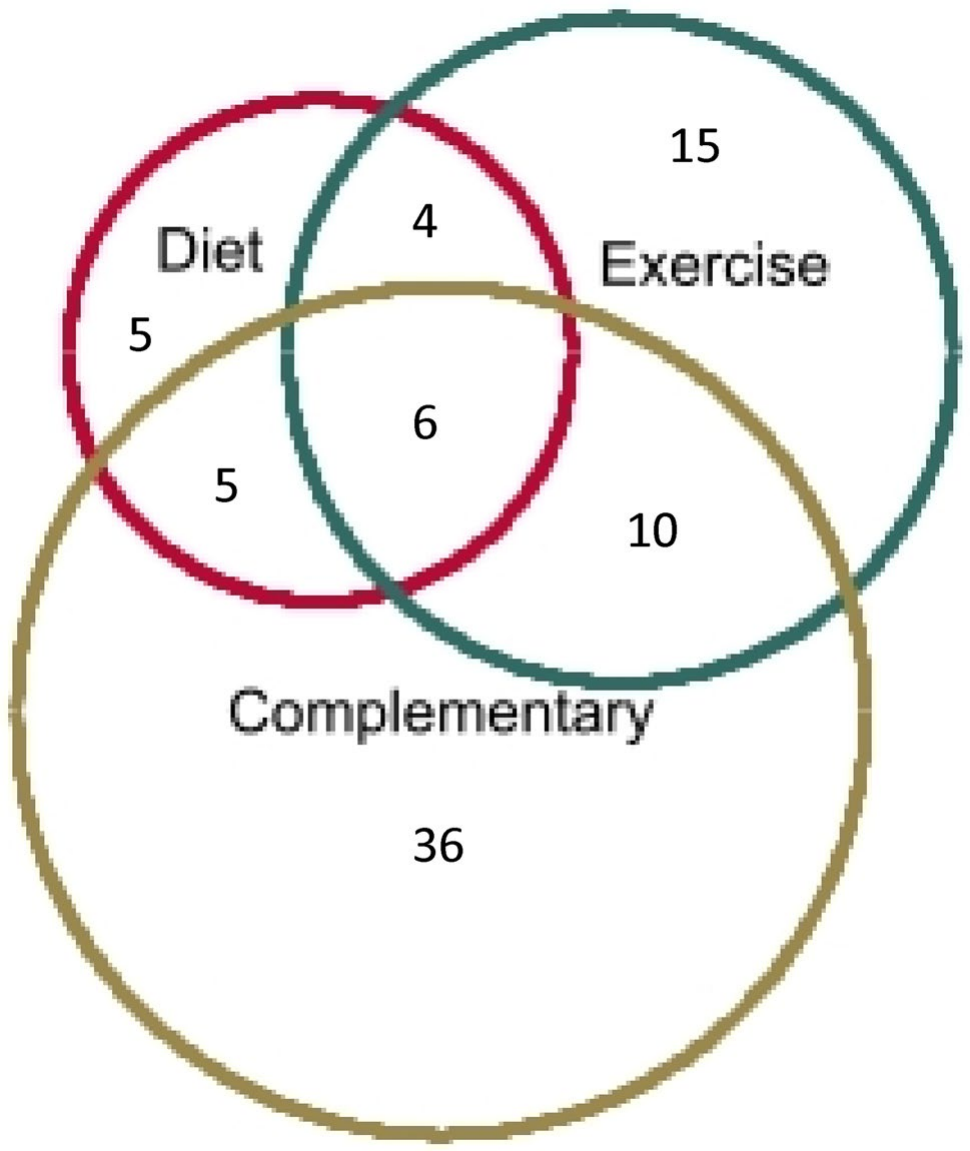
Venn diagram showing overlap of diet, exercise and complementary therapy users with numbers representing *n*

The most utilized type of therapies was complementary therapies, which 57 respondents reported, followed by exercise therapies (35 users). There was significant overlap between groups (Fig. 1), with 19 using any two of combinations of diet/exercise/complementary therapies and 6 utilising all three types. In total, 50 participants (25.4%) reported being advised on PMR specific exercises by a healthcare professional, 34 reported receiving exercises from a physiotherapist (17.7%), 18 from a general practitioner (9.1%) and 12 from a hospital physician (6.1%) and some from multiple sources. Of the 50 participants advised on PMR specific exercises, only 13 (26.0%) reported utilising exercise.

The most popular non-pharmacologic therapy reported was massage with 29 users (14.7%), followed by change in exercise with 26 users (13.2%). Therapies less frequency utilized include Alexander technique (two users), homeopathy (five users) and aromatherapy (six users). In terms of perceived helpfulness, massage had the highest proportion who perceived it to be helpful (25/29, 86%), followed by change in exercise (22/26, 84.6%).

Long-term outcomes were broadly similar between users and non-users of non-pharmacological therapy, with no difference in long-term pain and stiffness scores, or prednisolone dosage at follow-up (Table 2). Users of non-pharmacological therapies reported more flares (33.3% vs 25.0%), less improvement in symptoms (71.3% vs 79.3%) and were less likely to report being able to raise their arms above their heads (79.0% vs 89.7%) compared to non-users at LTFU. A significant number of participants in all groups reported prednisolone use at LTFU although fewer users of non-pharmacological therapies reported using prednisolone (23 people (28.4%) users vs 45 (38.8%) non-users).

**Table 2:** Long-term outcomes of users and non-users of non-pharmacological therapies

Outcomes	No therapy (*n* = 116)	Any therapy (*n* = 81)	Increased exercise (*n* = 35)	Diet/Weight Loss (*n* = 20)	Complementary users (*n* = 57)	Recommended exercise (*n* = 50)
Follow-up pain score NRS [adjusted mean (95% CI)]	2.5 (2.0–3.0)	3.3 (2.7–4.0)	3.2 (2.3–4.1)	4.7 (3.5–5.9)	4.5 (2.8–4.2)	3.5 (2.8–4.3)
Follow-up stiffness NRS [adjusted mean (95% CI)]	2.7 (2.2–3.2)	3.7 (3.1–4.4)	3.4 (2.4–4.4)	5.3 (4.1–6.6)	3.8 (3.1–4.6)	3.9 (3.1–4.7)
Raise arms above head today? *n* (%)	104 (89.7%)	64 (79.0%)	30 (85.7%)	15 (75.0%)	45 (78.9%)	38 (76.0%)
Current use of prednisolone, *n* (%)	45 (38.8%)	23 (28.4%)	12 (40%)	9 (45.0%)	11 (19.3%)	18 (36.0%)
Current prednisolone dose [adjusted mean (95%CI)]	2.7 (1.6–3.8)	1.8 (0.6–3.1)	2.0 (0.1–4.0)	1.9 (-0.8 – 4.6)	3.1 (2.2–4.1)	1.9 (0.2–3.5)
Symptom Improvement *n* (%)	92 (79.3%)	58 (71.3%)	24 (68.6%)	9 (45.0%)	41 (71.9%)	32 (64%)
Any flares, *n* (%)	29 (25.0%)	27 (33.3%)	11 (31.4%)	10 (50%)	20 (35.1%)	15 (30%)

## Discussion

Glucocorticoids remain the mainstay of treatment for PMR, despite adverse events associated with prolonged use. This study demonstrates that more than 5 years after diagnosis a large number of patients are still taking glucocorticoids. We have demonstrated that the use of non-pharmacological therapies in PMR is common with 41% utilising at least one therapy and 29% at least one complementary therapy. Many non-pharmacological therapies were perceived to be helpful, with most participants reporting change in exercise (85%), massage (86%) and hydrotherapy (81%) as being helpful, although none of the non-pharmacological therapies utilised were associated with significant difference in pain, stiffness or prednisolone dosage at LTFU; however, a cohort study is not the most effective at investigating such associations.

Overall, 81 out of 197 participants used at least one non-pharmacological therapy in the management of their PMR, with many participants utilising more than one therapy. Users of non-pharmacological therapy tended to be younger (69 vs. 74) and more likely to be female (74% vs 56%) in line with the general population [12]. Compared to studies of people with, rheumatoid arthritis and osteoarthritis rates of non-pharmacological therapy use is lower (41% vs. 98% and 99%, respectively) [10], which may reflect a better developed evidence base in other conditions. The demographics of the osteoarthritis patients surveyed were similar in terms of age (70 vs. 72) and gender (72% vs. 64% female); however, the rheumatoid population were significantly younger (57 vs. 72) and more likely to be female (80% vs. 64%), In comparison, research into non-pharmacological therapies for PMR is sparse, with just two non-randomised small scale trials into Chinese herbal therapies reported in the literature [13, 14].

Whilst the most commonly reported type of non-pharmacological therapy was complementary therapies, this was a combination of a variety of different interventions. Despite guidelines advocating exercise in PMR only 35 (17%) report using exercise to manage their PMR and 50 (25%) report receiving specific exercise advice from a clinician. Of those who reported using exercise to manage their PMR, 85% reported benefit. Although there is a gap in the evidence to support the use of exercise for PMR specifically, given its effects on increasing skeletal muscle mass and function, reducing inflammation and improving joint function in rheumatoid arthritis, resulting in long-term improvement in pain, stiffness and overall functioning [15]. Additionally, exercise is well known to reduce weight, improve glycaemic control and increase bone density, and, therefore, may counteract the side effects of glucocorticoid therapy in PMR. In our study, there was little difference in long-term outcome between users and non-users of exercise therapies; however, interpretation is limited as data was not collected on length of use, exercise intensity or timing of exercise relative to disease onset or reporting of outcomes. The overall association between exercise and PMR outcomes is not clear and requires further research.

Despite the paucity of evidence, the most recent EULAR guidelines recommend an “individualised exercise programme” should be considered for PMR patients [9]. In our cohort, only 50 participants (25%) were advised to perform PMR specific therapies by a healthcare professional, and of these just 13 (26%) reported undertaking a change in exercise. Barriers to exercise identified in rheumatoid arthritis patients include uncertainty over which exercises may be beneficial and fear that exercise may worsen joint health and therefore symptoms [15]. Further research into barriers to exercise use in PMR patients, and clinicians’ perspectives on exercise are needed to tailor appropriate patient information and improve rates of exercise use. The larger number of people reporting using exercise to manage their PMR, despite it not having been recommended by a health professional suggests this may be appealing to patients.

The reported use of physiotherapy within the cohort was low, with only 17.3% given exercises from a physiotherapist. In comparison, for adhesive capsulitis and rotator cuff tears in the UK, 71% and 77% of GPs refer patients to physiotherapy, respectively [16]. However, lack of commissioned pathways and local resources may be a factor in the low use of physiotherapy. The Chartered Society of Physiotherapy has highlighted increased workloads and reductions in physiotherapy services across the UK, with large regional disparities [17]. Other barriers to physiotherapy access may also have a role. A study of physiotherapy referrals for hip/knee osteoarthritis in the UK, demonstrated that only 41% of potentially eligible patients were recommended physiotherapy by their GP, and of these 17% then did not attend physiotherapy [18]. Given the high number of MSK comorbidities in PMR patients [19, 20], physiotherapists are well placed to provide safe, effective and individualised exercise in this heterogenous patient group, therefore further research into the role of physiotherapy in PMR is needed.

The motivation behind the use of non-pharmacological therapies is complex. Research from rheumatoid arthritis demonstrates factors including treatment dissatisfaction, poor relationship with a healthcare provider, and perceived benefit of alternative therapies drive their use [21]. In our cohort, a significant number of participants had little improvement in stiffness or pain, suffered from disease relapses and remained on prednisolone at long-term follow-up. It is unclear whether is long-term prednisolone use is related to exercise use and if so, whether continued need for glucocorticoids drives the desire for alternative treatments or vice versa.

## Conclusion

The use of non-pharmacological therapies in patients with PMR is common, with many patients utilising multiple therapies. Exercises were used by 17% and recommended to just 25% of the cohort. The lack of current evidence-based therapies beyond glucocorticoids is detrimental to long-term patient outcomes and may result in dissatisfaction in overall care. Further research is needed to identify which non-pharmacological therapies provide benefit to patients with PMR.

## Declarations

### Data availability

Keele University is a member of the UK Reproducibility Network and committed to the principles of the UK Concordat on Open Research Data. The School of Medicine and Keele Clinical Trials Unit have a longstanding commitment to sharing data from our studies to improve research reproducibility and to maximise benefits for patients, the wider public, and the health and care system. We encourage collaboration with those who collected the data, to recognise and credit their contributions. The School of Medicine and Keele Clinical Trials Unit make data available to bona-fide researchers upon reasonable request via open or restricted access through a strict controlled access procedure. The release of data may be subject to a data use agreement (DUA) between the Sponsor and the third party requesting the data. In the first instance, data requests and enquiries should be directed to medicine.datasharing@keele.ac.uk.
